# The neuroaesthetics of prose fiction: pitfalls, parameters and prospects

**DOI:** 10.3389/fnhum.2015.00442

**Published:** 2015-08-03

**Authors:** Michael Burke

**Affiliations:** Rhetoric, University College Roosevelt, Utrecht UniversityMiddelburg, Netherlands

**Keywords:** literary discourse processing, memory, neuroaesthetics, perception, prose, rhetoric, style, style figures

## Abstract

There is a paucity of neuroaesthetic studies on prose fiction. This is in contrast to the very many impressive studies that have been conducted in recent times on the neuroaesthetics of sister arts such as painting, music and dance. Why might this be the case, what are its causes and, of greatest importance, how can it best be resolved? In this article, the pitfalls, parameters and prospects of a neuroaesthetics of prose fiction will be explored. The article itself is part critical review, part methodological proposal and part opinion paper. Its aim is simple: to stimulate, excite and energize thinking in the discipline as to how prose fiction might be fully integrated in the canon of neuroaesthetics and to point to opportunities where neuroimaging studies on literary discourse processing might be conducted in collaborative work bringing humanists and scientists together.

## The Challenges Posed by a Neuroaesthetics of Prose Fiction

Neuroaesthetics is a flourishing field of investigation. Many influential book length studies have been conducted in recent years in testament to this (Skov and Vartanian, 2009a; Shimamura, 2013; Chatterjee, 2014; Lauring, 2014, etc.). Similarly, important volumes have been written on related topics such as, the neuroscience of aesthetic experience (Starr, 2013), aesthetic responses and evolved human behavior (Davies, 2012), what love and art reveal about the brain (Zeki, 2009), where art comes from and why (Dutton, 2009), the neuroaesthetics of art history (Onians, 2007), etc. Concurrently, a number of insightful articles on neuroaesthetics have been published in leading journals (Chatterjee, 2004, 2011; Kawabata and Zeki, 2004; Leder et al., 2004; Vartanian and Goel, 2004; Cupchik et al., 2009; Kirk et al., 2009; Croft, 2011; Huang et al., 2011; Ishizu and Zeki, 2011; Nadal and Pearce, 2011; Umilta et al., 2012; Vessel et al., 2012, 2013; Chatterjee and Vartanian, 2014, etc.). The main focus of all these works has been on the neuroaesthetics of the visual and auditory arts, namely, painting, dance and music. But what of literary prose? Where is the robust and extensive body of work on the neuroaesthetics of fiction? Even in the most recent publication on neuroaesthetics (Huston et al., 2015), literature plays no significant role in any of the 25 chapters, which again are primarily devoted to the neuroaesthetics of painting, dance and music. There has been some associative neuroaesthetic work done on prose fiction (Miall, 2009) but this has not dealt with the neuropsychological research on literary reading head on, simply because no such body of work existed at the time of writing. It could be cogently argued that six years later, it still largely does not. In what follows, the pitfalls, parameters and prospects of a neuroaesthetics of prose fiction will be reviewed.

## Literature: A Neurally Indiscernible Art Form

A central question directs the discussion in this article: why does neuroaesthetics not do literature? An answer could be it is simply too difficult and too challenging for researchers to access the effects of human interfaces with literary art forms. Painting, music and dance are regularly studied in neuroaesthetics because the effects can be relatively easily measured. They are all what will be termed here the discernible arts in that they are readily perceptible. Literature is not readily perceptible. As such, it can be termed an “indiscernible” art form: an art form that does not have a seemingly straightforward, one-to-one sensory fit. In viewing a painting, for example, light will bounce off the object and strike the retina. Initial neural activity will involve that retinal input moving to the back of the eye where it gets transduced into electric impulses, which then travel via parallel pathways to posterior parts of the cortex and then on to the prefrontal cortex. It is here that the rest of the neural activity gets ushered in as features are extracted and processed in the parietal and temporal cortices. Based on this, it can be said that the sensory input of painting and art primarily makes use of the occipital lobe and that there is a relatively straightforward route from an art object in the world—or indeed any object in the world that generates aesthetic feelings in the beholder (see Skov and Vartaniain, 2009b)—to the visual cortex in the brain. The same can be said of music in relation to the ear and the auditory cortex and general temporal lobe activity.

How does this equate to literature and to literary reading? Literary reading is mostly, and in most forms, a visual act. The type of visualization it employs, however, is different to the vision involved in pictorial art interfaces. Unlike viewing a painting in the world, there is no discernible object out there for light to bounce off and strike the retina. There are just words on the page or screen: those small, simple, culturally-determined semiotic signs. Indeed, light still strikes the word and letter shapes, it enters the retina, the input gets transduced into electric impulses, which then travel in a parallel processing fashion to the visual cortex and then on to the pre-frontal cortex. But the words, even if they appear in the visual cortex, as has been recently argued (Dehaene et al., 2002; Dehaene and Cohen, 2011), must be decoded. This is a process that involves semantic neural solutions. It is the meaning of the word form that matters and its immediate and subsequent context, not the form itself. There is also the fact that written literary input is also represented in mental imagery. Literary reading induced mental imagery may be mainly fleeting and indistinct, but when it appears it is robust and recyclable (Burke, 2011: 56–85). Moreover, it is held dear and valued by many avid readers. It is as if it has become part of who they are; an implicit and integral part of their beliefs-values-attitudes system. In short, literary reading is primarily visual, but is it not the readily accessible kind of vision that painting and other visual art forms offer. It is not directly available, in a simplistic one-to-one fashion through one of the major sensory organs.

So does this make the neuroaesthetics of fiction a less attractive object of study compared to the neuroaesthetics of painting, music and dance? The answer to this question is no, not at all. It does, however, make it more complex, as one must be explicit about what is being measured, how and why. Concepts like the pre-reading mood and the actual time and spatial location of the reading event are not insignificant factors when it comes to an embodied facilitation of suitable positive emotions for fulfilling literary interfaces (Burke, 2011). As Varela et al. (1991: 173) first noted in their pioneering embodied cognition work, cognition depends to an important extent on the kinds of experiences that our bodies undergo in cultural contexts.

A further complicating question can be posed whether or not literature relies primarily on visual or auditory stimuli? A first reaction might be to say that it is a visual process. But is it that simple? There are at least three reasons why this conclusion seems premature. First, there is the argument just made: that the object being perceived is not directly “seen”. Literature may paint pictures in the mind but literary language is no painting. There is no direct input in the traditional sense. Those culturally determined semiotic signs have to be transduced in the visual cortex to produce indistinct, yet robust mental imagery. The visual cortex is centrally involved but it is a “deferred” rather than direct kind of visual processing that takes place. Second, reading can be done without sight. Braille is the tactile route to literary pleasure. The sighted and the sightless both enjoy reading literature. Avid readers can engage in literary discourse processing without the need for visual stimuli. From a processing perspective, experiments have shown that the tactile processing pathways usually linked in the secondary somatosensory area are redirected in blind subjects to the ventral occipital cortical areas formerly assigned to visual shape discernment (Sadato et al., 1998). Third, an increasing number of studies are showing just how active the auditory cortex is during silent acts of literary reading (Yao et al., 2011, 2012; Perrone-Bertolotti et al., 2012; Eerland et al., 2013; Petkov and Belin, 2013; Brück et al., 2014). Indeed, the auditory cortex might even be just as active as the visual cortex, even though there are no external sounds in the world to be appraised via the ear during the reading event. These three observations therefore, when taken together, suggest that the claim that literature is a purely visual stimulus may be too simple and rather imprecise.

The question of whether reading narratives differs from hearing them is no longer introspection. A fMRI study was conducted by Regev et al. (2013) which addressed the question of how similar is the neural processing of linguistic content when the same real-life information is presented in spoken and written form. Looking at response times elicited by spoken and written narratives the researchers found that early visual areas responded selectively to the written version, and early auditory areas to the spoken version of the narrative. Moreover, many higher-order parietal and frontal areas demonstrated strong selectivity, responding far more reliably to either the spoken or written form of the narrative. By contrast, the response time courses along the superior temporal gyrus and inferior frontal gyrus were remarkably similar for spoken and written narratives, indicating strong modality-invariance of linguistic processing in these circuits. The authors conclude that these results suggest that our ability to extract the same information from spoken and written forms arises from a mixture of selective neural processes in early (perceptual) and high-order (control) areas, and modality-invariant responses in linguistic and extra-linguistic areas.

It is also important to note that literary reading is not an indiscernible art in all its forms. The perceptibility operates on a continuum. A first example of this is when stories are read aloud to another person. Such cases are primarily auditory and thus directly discernible in the parietal cortex. The amount of emotion experienced by the listener during the act of being read aloud to also depends on who is actually doing the reading and the relationship between the reader and hearer. This too operates on a continuum with at the one end say a mother or father reading to her/his child and at the other end people listening (and watching) a story being read to a general audience by a stranger via a YouTube platform. A second example is when a reader reads a story aloud, either to him/herself or to another person or persons. In such cases, literature occupies an area in between the discernible and indiscernible. An interesting addition to this is that the voice that the reader hears while reading aloud is not the same voice that the audience that is being read to hears. The voice that the audience hears is much higher. It is also the real voice. The voice that a reader hears, which is of course the same voice he/she hears when speaking generally, is much lower and does not exist in any real sense. The voice sounds lower because it has traveled internally, as it were, from the vocal cords, through the bone of the jaw and skull and into the cochlea. Normally, sounds travel externally through space and strike the ear drum which is the start of the auditory processing procedure. A third example is when a reader reads literature silently, as is the norm nowadays, although this has not always been the case (Manguel, 1996). In such cases, it is an indiscernible art. The point here is that in the field of neuroaesthetics, and indeed more generally, one cannot simply say that literature is visual procedure. Literary discourse processing is a fluid concept in many ways. One needs to first define which kind of literature one is speaking about and proceed from there.

## What is Literary Reading?

Literature has often been circumvented as an art form in neuroaesthetic studies. A question that has to be considered as a plausible reason for this is “has this decision been grounded on an external reality”, namely, that literature is generally considered a minor art in mainstream aesthetics, as opposed to the likes of painting, music and dance? The idea of comparing the value and status of the various art forms goes right the way back to antiquity and specifically to the muses. According to Hesiod, the muses were the nine daughters of Zeus and Mnemosyne (the goddess of memory) who resided on mount Helicon in Boeotia, Greece. The daughters were called Clio (history), Euterpe (lyric poetry), Thalia (comedy), Melpomene (tragedy), Terpsichore (choral dancing), Erato (love poetry), Polyhymnia (sacred music), Urania (astronomy) and Calliope (epic poetry). They were all allotted a different object that represented their particular art. This could be, for example, a flute or a lyre or a writing tablet. Calliope, the muse of epic poetry, is said by Hesiod (1988: 5) to be the most important “chief among them all” see also Skarsouli, 2006). The concept of memory is central for all nine muses, a trait they had inherited from their mother. The main job of the muses was to inspire the arts, in particular music and poetry which were considered two sides of the same coin. This suggests that in antiquity at least literature and poetry were not considered minor arts.

Aristotle (1965) also regarded literature highly. He taught poetics at the Lyceum and wrote on the subject in his model of the narrative structure of tragedy. His investigations led to his famous theory of catharsis. The central idea is that an audience at the theatre would experience intense emotions vicariously, as it were, and that this is important for their everyday functioning as citizens in society. This is achieved in the theatre by observing how seemingly unwarranted events of great magnitude overcome a favored, yet fated, protagonist. An audience first pities his or her seemingly underserving plight. The pity then turns to fear as a number of hypothetical scenarios are non-consciously loaded into the conscious mind of the viewers along the lines of “I hope that something like that never happens to me or my loved ones”. Longinus, a Roman scholar, developed a theory of the poetic sublime, claiming that there are five sources of sublimity. The most important according to Longinus is the ability to form grand conceptions, to have inspirational and creative ideas: the grandeur of thought. The second pertains to the stimulus of and the capacity for powerful and inspired emotions. Both these are essentially cognitive and innate. The next two sources of the sublime are also related. These however pertain not to innate qualities but to application: the extrinsic notions of skill and art are prominent here. The first of these two is the ability to create truthful examples of two types of rhetorical figure: figures of speech and figures of thought. The second is the capacity to create noble diction. What these two categories have in common is their focus on word choice, imagery and style. The fifth and final source of the sublime, which embraces all of the previous four, is the total effect resulting from formality and elevation: what might be termed “the dignity of composition” (Longinus, 1965: 108–109). The notion of the sublime was dealt with again much later in the eighteenth century by statesman and philosopher Edmund Burke. In his work *A Philosophical Inquiry into the Sublime and Beautiful* published in 1757, Burke commented on how words, both eloquence and poetry (i.e., rhetoric and poetics), are much more capable of making profound impressions on individuals than any of the other arts, including the more visual/pictorial arts. Writing on the effects of words, Burke (1998: 190 emphasis as in original) states “if words have all their possible extent of power, three effects arise in the mind of the hearer. The first is the *sound*, the second the *picture* or the representation of the thing signified by the sound; the third is the *affection* of the soul produced by one or by both of the foregoing”. The question arises what are the effects of literary words that can be observed, recorded and measured in the brain? In light of these observations, from Hesiod, through Aristotle and Longinus to Burke, it can be suggested that the reason more time is not spent on exploring literature in neuroaesthetics is not because it is considered a lesser art form. Another plausible reason as to why neuroaesthetics has not invested much in literature could have something to do with the historically difficult relationship between aesthetics and literary studies. Unlike art and music, literature—and in particular literary theory—has spent decades focusing on a range of aspects other than a reader’s evaluation of the beauty of a text. While this might not be the case for poetry, it most certainly is for prose fiction. Arguably, a coming together between modern literary theory and aesthesis could open the door to more work being conducted in the neuroaesthetics of fiction.

To understand more fully the deferred and indiscernible nature of literary discourse processing one needs to appreciate what reading actually is. Firstly, reading is a profoundly unnatural activity. Human brains were not designed to read. The brain, however, is adaptive and, as a result, that most artificial of activities, reading, and especially literary reading, is not only second nature to many people in the world today, it is also an important, almost spiritual, part of their daily lives. Learning to read changes the make-up of the brain. In particular it changes the cortical networks for vision and language (Dehaene et al., 2010). Although at a very early stage, neuroscience is starting to unravel the mysterious act of reading. This new science of reading recognizes that our brains, which have we have inherited from our early ancestors—and the processing networks that are operational within them—have been recalibrated, as it were, to the task of word recognition. The claim here is that neuronal networks have been “recycled” for reading (Dehaene, 2009). Brain scanning experiments have recently shown that reading systematically activates a specific area of the visual cortex normally designated for object and face recognition: the left lateral occipito-temporal sulcus (Cohen et al., 2000; Dehaene et al., 2001, 2002; Cohen and Dehaene, 2004; Dehaene, 2009; Dehaene and Cohen, 2011). This brain site has been dubbed the “visual word form area” (VWFA). Lesions in the VWFA cause pure alexia in individuals, resulting in severe reading problems, while other language related skills, such as speaking, comprehension and writing are typically left functioning normally. The VWFA hypothesis has popular support. However, it also has its detractors (Price and Devlin, 2003, 2011).

Reading is essentially a motivated, goal-directed activity. This is especially the case with regard to literary reading, where the goals are largely the attainment of both pleasure and knowledge, as Horace put it, the goal of literature/poetics is to teach and delight (*aut prodesse volunt aut delectare poetae*). The pleasure aspect is arguably the most significant in most readers and certainly in most avid prose readers. Avid readers, desire literature, need literature, love literature. The skeletal essences of favored literary fragments are always with avid readers, undulating in the non-conscious backwash of their oceanic minds. The times of the day, week or year that they choose to read and also the places and positions they subconsciously elect to place their bodies into to read are selected with non-conscious fastidiousness for maximal hedonic impact. Avid readers are self-primed, as it were, through prior habitual experience to enter the semi-conscious state of literary discourse processing.

Many readers in such engaged reading states may be transported to other worlds. Transportation is the general idea that when readers get “lost in a story” their attitudes and intentions alter to reflect that story. Early work on narrative transportation theory was conducted by Gerrig (1993). Gerrig’s ideas on transportation have since been extended and deepened. Green (2004), for example, has focussed on the ability of stories to transport readers. This is seen as a mechanism of narrative impact. In her study, participants read a story and then rated it for transportation, perceived realism and story relevant beliefs. Transportation was positively correlated with perceived realism Moreover, subjects with previous knowledge or experience pertinent to the main themes of the story showed higher levels of transportation into the narrative.

In my own work I have theorized on the notion of what I have termed reader disportation (the word disportation (disport) embodies the two concepts of movement and positive emotion). As yet, no substantial experimental work has been conducted to falsify this theory. The unnatural and unavoidably intrusive nature of the laboratory setting is a formidable challenge to be overcome in the research design of any disportation study. Disportation is concerned with felt motion, not actual motion. It is a heightened emotive state that occurs in affectively-engaged individuals while reading literature. It is characterized by a distinct feeling that a reader undergoes for a few seconds whereby a person feels that he/she is in motion, even though this is not the case. It is a felt motionless movement through space. In many ways, disportation is a simulated, embodied affective-cognitive event that must include mirror-neural activation (Burke, 2011: 231–232). The “arousal” followed by “calm” pattern that is inherent to disportation is based in the pioneering work of Berlyne (1960, 1971, 1974) and especially in his notions of “tension and release” and “boost and jag”.

Disportive and disportive-like episodes during reading have been reported by readers. For example, the writer Ginsberg (1966: 40) famously wrote how upon reading William Blake his body suddenly felt light and he felt vibrations which flowed into sensations of awe, understanding, wonder and surprise. He added that it felt like the top of his head had come off and that the universe was intermingling with his brain. The scholar Kaplan (1993: 58) has written about what happened to her as she finished reading the closing lines of *The Great Gatsby*. She tells of how it was afternoon and the sun was coming in through large window which looked out onto the mountains in the distance. The sun which was shining into her eyes made her realize that she has stopped reading and was crying. In addition to such reflective self-reporting, I have conducted my own (qualitative) experiment (sample size 36). One of the open questions that the literary reading questionnaire, asked subjects is whether they were able to recall finishing a much enjoyed novel and if so whether they could recall their feelings at the time. Although most subjects reported having experienced no disportive effects during reading, some commented that they had and that at such moments they had felt either “climactic”, “euphoric”, “very emotional” or “excited”. Indeed, one subject reported that he/she “felt a sort of release” (see Burke, 2011: 176 for the data). Comments like this are not markedly dissimilar to the self-evaluation reports of Ginsberg, Kaplan and others. What they suggest is a kind of reader epiphany (not be confused with the kind of “Joycean” literary epiphanies, which overcome characters in novels). A change in understanding also takes place, just as it does with aesthetic appreciation in general. Indeed, one of the subjects in the experiment reported his/her disportive experience as “Very emotional. Disappointed and euphoric at the same time. But also calm and focussed (in a Zen sort of way)—taking it slow.”

Transportation and disportation differ. Transportation entirely takes place in the flow of narrative events: in what can be seen as the implicit, non-conscious, swift narrative processing of backgrounded, mainly “story advancing”, textual elements. The lead up to disportation starts in the same non-conscious domain as transportation, but the event of disportation itself can only take place once the explicit, slow narrative processing of foregrounded, chiefly, “story enriching” rhetorical textual elements have been reached. This is neither fully non-conscious nor fully conscious, but somewhere in between, and probably functioning on a continuum flowing in and out of conscious appraisal. This blend of non-conscious and conscious elements is important as it sits between two different views. Studies have shown that foregrounding appears to occur early in responses. This means that, perhaps surprisingly, it does so largely outside of conscious awareness (Posner and DiGirolamo, 2000; Eckstein and Friederici, 2006). This claim falls within the general framework of what is known about event related potentials such as N400 (Kutas and Federmeier, 2000, 2011) and P600 (Hagoort, 2003). However, as Vartanian (2009: 261) has argued, when we perceive and interact with an artwork we can become conscious of the experience of pleasure. To my mind this is wholly accurate. What the act of disportation then offers us is a kind of bridge between what I have previously termed “affective cognition” and “cognitive emotion” which together lead to “oceanic cognition” (Burke, 2011). The idea of oceanic cognition is that the communicative traits of literary reception (and probably also of literary production) are not located in distinct anatomical brain areas that converse unidirectionally, but rather that these are distributed blends and combinations of basic multidirectional neural processes. The central idea underlying the oceanic cognition theory complies with the widely accepted “diffuse neural network” idea of processing practices that take place in the brain.

The hedonic moments of reading experienced during disportation are largely unpredictable and can appear at any time in a literary text, as they rely in part on non-conscious memory, which is often autobiographical. However, highly crafted, strategically placed networks of linguistic rhetorical style, (placed consciously or otherwise and often concentrated at the end of a story) such as repetition, parallelism and deviations of rhetorical figures such as schemes and tropes, combined with themes, can help prompt and stimulate such hedonic, disportive episodes. Such occurrences, although rare, are always accompanied by brief yet intense emotion. There is also often a sense of felt movement or motion, a rising up followed by a descent. Motor and proprioceptive and vestibular brain regions are engaged during such episodes. The cerebellum plays a central role here. A distinction is generally made in humans between conscious and unconscious proprioception. Conscious proprioception is communicated by the posterior column-medial lemniscus pathway to the cerebrum (Fix, 2002), while unconscious proprioception is communicated primarily via the dorsal spinocerebellar tract and ventral spinocerebellar tract to the cerebellum (Siegel and Sapru, 2010: 263). Disportation would appear to make use of both systems. Style and memory play a critical role in such disportive, and indeed all, aesthetic experiences (see Skov, 2009: 10). Following Ishizu and Zeki (2011), and given the aesthetic nature of disportive events, it can be plausibly argued that at some stage the medial orbitofrontal cortex might be involved in acts of disportation—exactly when this takes place in the process remains as yet unclear.

## What Work has been done in Literary Neuroaesthetics?

Neuroaesthetics is the study of the procedures that motivate aesthetic behavior (Skov and Vartaniain, 2009b: 3). It is about the identification of aesthetic functions and the investigation of their neurobiological causes. No explicit work has been conducted on the neuroaesthetics of fiction (Miall, 2009: 233). Significant related work has, however, been conducted on the brain and literature by Jacobs and his colleagues (Jacobs, 2011, 2014, 2015a,b; Schrott and Jacobs, 2011; Altmann et al., 2012, 2014). Interestingly, it is not labeled neuroaesthetics of fiction but rather neurocognitive poetics. Jacobs’ neurocognitve poetics model of literary reading (Jacobs, 2011, 2014, 2015a), is a dual system model that hypothesizes how poetic texts are read and processed. It models processes at three different levels: neuronal, affective-cognitive and behavioral. The first, largely implicit, system hypothesizes a fast, automatic route that deals with background elements in the text. The second, largely explicit, system hypothesizes a slow route that deals with foregrounded elements in the text. The fast route is said to account for a number of important features of literary reading, including: (i) the above mentioned narrative transportation theory; (ii) the activation of situation models; and (iii) the earlier mentioned experiencing of fictional emotions in the Aristotelian sense of catharsis such a pity and fear. The slow route is said to be operational in aesthetic processes supported by schema adaptation, artifact emotions and basic ludic neural systems. At a neuronal level the fast, background route, which involves implicit processing, is said to mainly employ the left hemisphere reading network, where non-aesthetic fiction feeling are evoked. Specific areas are the anterior temporal lobe, which has been cited as an important area for proposition building, the posterior cingulate cortex, the ventral precuneus and the dorsal medial prefrontal cortex (Jacobs, 2015a: 148). The slow foreground route, involving explicit processing, mainly employs the right hemisphere reading network. At a behavioral level, the fast background route involves fluent reading, that is, short fixations, large saccades and low affect ratings, while the slow foreground route involves slowed reading, that is, long fixations, small saccades and high affect rating (Jacobs, 2015a: 142).

Reflecting on this neurocognitive poetic model of literary reading, it should be said that it does have a distinct aesthetic aspect to it, in particular with regard to the explicit “slow route” of processing. The central research question, however, in neurocognitive poetics is not that of neuroaesthetics which is “what are the neural mechanisms that allow us to experience beauty in art?” Neurocognitive poetics also has thus far not explored the relationship between their own models and, for example, Zeki’s findings with regard to beauty and the medial orbitofrontal cortex. Jacobs and his colleagues, however, have conducted some compelling work with regard to aesthetics and proverbs (see Bohrn et al., 2012). While proverbs are not the same a prose fiction, the selection of such idiomatic expressions for experimentation is intriguing and commendable. Following up on this, in a recent fMRI study on poetic discourse, Zeman et al. (2013) found that highly foregrounded poetic texts, that is poetic texts that exhibited literariness features, exhibited more brain activity than less foregrounded texts. Activity was predominantly present in left-sided regions, such as the precentral gyrus and areas of the basal ganglia. This study can be seen as firmly in the domain of a neuroaesthetics of poetry. Interestingly, Zeman et al.’s (2013) finding does not match Jacobs’ hypothesis, which situated foreground processing in the right half of the brain. It would be an interesting undertaking to blend Jacobs’ model with Leder et al.’s (2004) earlier, and more general, model of aesthetic appreciation and judgement to see whether taken together these hypotheses hold.

In his recent article Jacobs (2015b) looks at methods and models for investigating the neuronal and cognitive-affective bases of the reception of literature. His starting point is the observation that despite there being a strong tradition in fields like poetics and stylistics, little is still known about how the brain creates and processes literary and poetic texts. He had previously argued that because discourse genres like literature and poetry succeed in succinctly and optimally combining thought and language and music and imagery, together with pleasure and emotion, stimulus material like this would be best suited, rather than other discourse genres, for helping to explain the complexities involved when human brains construct the world in and around people (Schrott and Jacobs, 2011). In short, if you want to get a grip on how the fluvial brain works, conduct experiments using literature. I have made a similar argument on the topic of the oceanic mind and literary reading (Burke, 2011).

In that study, I addressed the question “what happens in the brains and bodies of avid, engaged readers when they sit down to read a work of fiction?” (Burke, 2011). This was done by applying theory and existing empirical experiments from cognitive neuroscience and by conducting primarily qualitative testing on a number of subjects. The theory of the oceanic literary reading mind maintains that there is a dynamic, free flow bottom-up and top-down phenomena termed “affective inputs”. These are pre-reading mood, the time and place of the reading event, literary reading induced mental imagery, the style/rhetoric of the discourse and the narrative themes of the text. These are all active during engaged acts of literary reading by avid readers. The theory contends that literary reading does not begin when eyes apprehend the words on the page or end when they leave off, rather, the mind, brain and body are actively reading both before and after the physical act of literary text processing starts and finishes. This is the “literary reading loop” (LRL) and is captured in the four interconnected stages of pre-reading, actual reading, post-reading, and non-reading. Pre-reading is that window of time just before eyes meet the page/screen. Post-reading is the period just after eyes have left the page/screen. Non-reading is the time in between reading events where a reader could be out for a walk, eating dinner at home or driving to work. The theory of the oceanic literary reading mind highlights the significance of unconscious “affective cognition” and implicit memory during acts of engaged reading, alongside the more conscious cognitive emotion and explicit memory. It taps into the idea of cross-cortical and cross-modal processing in the brain and it models these ever-shifting, dynamic brain processes as “oceanic cognition” (Burke, 2011, 2015).

Other relevant work has also been conducted in this area. Hogan (1996, 2003, 2013), for example, has published widely in the field. In his 2013 article he observes that in the novel *Ulysses*, Joyce explores both parallel and serial thought processes. Hogan argues that this can help science to better understand the idea of cognitive parallelism, the notion that neural thought is not a serial process. Harmony and counterpoint are two notions at the heart of Hogan’s study. These can also been seen, albeit in a more foregrounding sense, in Armstrong’s (2013) neural-hermeneutic study on how literature plays with the brain through the experience of harmony and dissonance. These, Armstrong argues, trigger oppositions that are central to the neurobiology of mental functioning. A similar argument is made by Starr (2013) who proposes in her study that aesthetic experience, including that of poetry, relies on a distributed neural architecture that includes memory, emotion, language and perception. It can be argued that there is a pattern emerging here that argues that the humanists should be more proactive in their relationships with scientists, as are Hogan, Armstrong and Starr. Most contemporary interaction is basically one-way traffic: the humanities take ideas from science and apply them in a humanities subject area. Within the framework of “cognitive literary science” the argument has been made that instead of literary studies perennially taking its prompts and inspiration from science for interdisciplinary projects, literature should be providing cognitive science with humanities theories for them to test (Burke and Troscianko, 2013).

A further inspiring empirically tested hypothesis that is ripe for neuroaesthetic investigation is Miall and Kuiken’s (2001) “defamiliarization-reconceptualization cycle” theory, which states that when a reader encounters stylistically foregrounded language in a text, his/her schemata may be inadequate for comprehension. As a result, the feelings evoked by the text facilitate alternative perspectives which can direct and motivate readers to search for new understandings. This reconceptualization can take place much later in the text (Miall, 2009). From a neurobiological perspective, primary responses to foregrounded passages will activate the amygdala (Robinson, 2005; see also Davidson et al., 2003). Following LeDoux (1998), the “quick and dirty”, “low road” to emotion will no doubt be involved here too.

## How Might Prose Fiction be Better Integrated in the Canon of Neuroaesthetics?

There is a rich body of empirical work conducted by literary scholars and psychologists coming out of organizations like the *International Society for the Empirical Study of Literature* (IGEL) that looks at literary reading processes, emotion and aesthetics. Many of these studies plausibly offer productive points of entry for literary neuroaesthetic work. In addition to the behavioral work done in quantitative literary empiricism, there is also the field of qualitative literary analytic study. Such structural and hermeneutic approaches in literary theory, which are much less well known in neuroscience, primarily because of their qualitative rather than quantitative approaches, include the study of rhetoric and style (and ultimately the study of foregrounding). This is more commonly known as stylistics, or literary stylistics or literary linguistics (see Jeffries and McIntyre, 2010; Burke, 2014). It is the structural aspect, whether it be the five canons of rhetoric or the various rhetorical schemes and tropes, that affords a promising point of entry for neuroaesthetics. In “The Potential of Style Figures and Meter in Neuroaesthetic Study” Section below, I report in detail on a previous study of mine concerned with how the structural nature of rhetorical schemes makes them aptly suited for neurocognitive rhetorical investigation. More generally, for now, the reason why structural approaches to prose fiction offer promising entry points into neuroaesthetics is that despite brain processes being contextual, distributed and dynamic, at their core they are also structured. Indeed, a number of studies have attempted to chart this underlying structural matrix (for example, Sporns et al., 2005) for their work on the human connectome and Kötter (2001) and Sporns and Kötter (2004) for their work on tools for exploring brain structure function relationships and the charting of motifs in brain networks. By following the heuristic of classical rhetoric as set out in its five canons, future studies could look for how the mnemonic, perceptual and emotive brain centers generate “materials”, how those material are arranged and thereafter stylized for attention and finally how they are rehearsed for delivery and ultimately “performed”. Such a rhetorical, structural approach to a neuroaesthetics of prose fiction could be more promising than say a narrative or immersion approach, because arrangement only accounts for one of the five canons of rhetoric. Put another way, narrative is just one small part of the classical rhetorical framework.

Cognitive poetics is a third field that warrants attention and in particular the kind of cognitive poetic work that is based on the cognitive psychology of memory, e.g., schema and script theory (Bartlett, 1932; Schank and Abelson, 1977; Rumelhart, 1980; Schank, 1983, 1999; Rumelhart et al., 1986). This cognitive psychological schema theory work has also been applied to literary stylistic analysis and theory forming (Cook, 1994; Emmott, 1997; Semino, 1997; Sandford and Emmott, 2012; Emmott et al., 2014). All these works should point to useful avenues of investigation into neuroaesthetics. There are many pertinent questions that neuroaesthetics could address. One of particular interest is does foregrounded literary language slow down the reading process, as has been claimed (see Miall and Kuiken, 1994; Burke, 2011; Jacobs, 2011, 2014, 2015a). This is a question that neuroaesthetics should be capable of answering with a combination of eye-tracking methods and EEG neural scanning technology.

The focus thus far has been on readers and readers alone, but maybe this is too narrow? Perhaps the question of whether or not a reader slows down when he/she encounters a foregrounded passage in a text might depend on the reader or on the story or on the context of the reading episode or on all three. The context or *kairos* of the reading event is paramount and is often overlooked. The kind of questions that could be addressed in neuroaesthetics are what are the neural processes that underlie aesthetic behavior when …, (i) a person reads prose outdoors instead of indoors; or (ii) a person reads while standing up instead of seated or lying down; or (iii) a person reads from an e-reader instead of from a paperback book; or (iv) a person has e-ink on his/her e-reader instead of a regular LCD screen. All these questions, which focus not just on visual and auditory aspects of literary reading but also on haptic and somatosensory phenomena, and many more, could be investigated with wearable EEG sensory headsets to test for electrical activity in the brain, combined with and galvanic skin response sensors to test for psychological or physiological emotive arousal.

## The Potential of Style Figures and Meter in Neuroaesthetic Study

There are plausibly two main impediments to conducting experiments in literary neuroaesthetics. The first and most significant of these concerns what might be termed “the neural mystery of meaning”. The challenge is summarized succinctly by Dehaene (2009: 111) who says that although we know quite a lot about meaning in the brain, for example, that it involves an extensive number of brain areas, the question of how meaning actually gets coded in the cortex “remains utterly mysterious”. Moreover, it must be remembered that in his reading studies Dehaene does not look at literary language at all, just at everyday written language. Imagine the challenge that reading literary fiction poses when it comes to how meaning gets coded in the cortex. How, for example, would neuroscience and neuroaesthetics account for the common literary phenomenon of intertextuality: the idea that the meaning of a text is shaped by a reader’s implicit knowledge and understanding of other texts? Put differently, how, and indeed where in the cortex—in both literary processing and literature production—does the brain code meaning for the intertextual input from such things as allusions, translations, quotations, parodies, pastiches, satires, etc. Intertextuality adds extra layers of meaning to texts. Indeed, those who produce literary texts purposely employ intertextual “echoes” of other texts in order to enhance and deepen a readers’ emotive reception of semantic input.

The second impediment is the nature of literary reading induced mental imagery. This kind of imagery may be indistinct but it is extremely robust. This was highlighted in a study by Sadoski et al. (1990) who had college students recall a short story and report the imagery experienced under three conditions. The recalling and reporting took place immediately after the story and again 48 hours later. One of their findings was that in the later examination the verbal recall of the events in the story had declined over the retention interval, while the mental imagery experienced while reading had not (see also Sadoski and Paivio, 2001). As Kuzmičová’s (2014: 275) puts it in a later study (in which she theorizes four different kinds of imagery) mental imagery is one of the commonest things that people will remember about their literary reading experience.

In my own work, based on qualitative data, I have argued that the backdrops against which mental imagery is staged while reading literature is often based on spatial, nonconsciousness (mainly) childhood memories. The robustness and recognizability of literary reading induced mental imagery contributes to their availability to non-conscious recall in future reading situations (Burke, 2011). More subjectively, ask anyone to go see the film version of their favorite novel and the chances are they will take issue with the imagery that is offered to them in the cinema: “that is not him”, “she doesn’t look like that” and “they don’t live there”. In short, the current challenge to neuroscience—pertaining to the elusive nature of meaning in the brain and the perplexity of literary reading induced mental imagery and its non-conscious and locative challenges—means that, for now, neuroaesthetics would be wise to explore more accessible ways into the brain. One such way would be to take as a starting point a rich vein of current neuroaesthetic study, namely, music (Tervaniemi, 2009; Brattico et al., 2010; Nieminen et al., 2011; Simoens and Tervaniemi, 2013).

Music and poetry have long been close, for they are rhythmic arts. As reported earlier in this article, even in ancient myth they are sisters Muses; two sides of the same coin. Since that time clear associations have been pointed to that exist between both literature and music (Neubauer, 1986; Meyer, 2002) and rhetoric and music (Bartel, 1997). At a macro-level, emotion is one of these links. Music and language also share some core brain areas like Broca’s region (Maess et al., 2001; Levitin and Menon, 2003; Patel, 2010). Musical and linguistic aptitudes have also been shown to be generally interlinked (Milovanov and Tervaniemi, 2011). Micro level links have not been as widely explored. It will be argued here that two of these should be, namely, style figures from rhetoric and poetic meter.

I have conducted some preliminary work on the rhetorical neuroscience of style figures (Burke, 2013; see also Bruhn’s (in press) application of the model). It was hypothesized that literary style elements—and particularly foregrounded units such as rhetorical schemes—can sometimes initially be in the mind and brain during engaged acts of literary reading, rather than always out there in the world; in effect, that seemingly bottom-up inputs might on some occasions initially be top-down. This assumption was then examined against a number of psychological and neurobiological storage and retrieval theories, both conscious and non-conscious (Baddeley and Hitch, 1974, 1988; LeDoux, 1998; Barsalou, 1999; Damasio, 1999; Baddeley, 2000; Bunge et al., 2000; Eichenbaum, 2011). Taking Barsalou’s (1999) perceptual symbols system theory as a model, it was further hypothesized that when a scheme (like a chiasmus, e.g., “falling faintly, faintly falling” from the end of James Joyce’s short story “The Dead”) enters the reader’s brain below the level of conscious appraisal, through the visual sensory channel, neurons in feature maps fire to create a representation that maintains both the words and the balance of the style figure (for a chiasmus, this is a basic AB–BA structure). Such representations mirror the original text fragment, e.g., in the example this would be disyllabic, alliterative, embodying a palpable sense of descent, etc. The essence of this scheme and the structural pattern is held in an associative neural area. This is probably in long-term memory with key involvement of the motor region to capture both the balance and movement that are inherent in style figures. The storage process will plausibly also involve a sub-vocal rehearsal region of short-term memory such as the articulatory loop. Any movement between long-term memory and buffer regions of short-term memory will be dealt with by the episodic buffer. By this stage, the words and the structure of the original text fragment are in a skeletal form and the whole is scarcely recognizable as “falling faintly […] faintly falling”. This process constitutes the “storage” part of the procedure.

The second, “simulation” stage works in two situations. The first, and most prevalent, situation is during a later engaged reading experience. Here, an enthused individual may be reaching the end of a much cherished reading experience when—following the idea that humans are disposed to play with and cherish patterns in literature (Boyd, 2012)—he/she may subconsciously long for the style figural, rhythmic rewards that were given to her/him by Joyce in his short story: that sense of balance, of perfection, of exactitude. Unbeknown to the reader, the skeletal essence of the “falling faintly […] faintly falling” pattern that has been captured in an associative neural area now fires, triggering the neurons in the feature map which also fire to produce a representation that is similar to the original in the feature map which was stimulus-driven through direct sensory input, but is less perfect in form. Style fragments flow into short-term memory to meet incoming sensory data from the new/ongoing reading experience.

The idea of longing for style figures falls within the framework of pattern recognition. At a cognitive level it is a matter of desire, and for some readers even one of addiction. Repeated literary reading generates a sensitivity for textual patterns, thus facilitating easier recognition in future literary interfaces (Hogan, 2009: 36). Hogan (2003: 20) has also written about how motifs in music can trigger memory. Style figures of repetition and parallelism act in similar motif-like ways.

It was argued here that the results of this “collision”, as it were, can vary. This depends to a large extent on the quality of the actual incoming data. On the page there may be no engaging style figures at all to be read, or they may be different style figures, or they may, in very rare cases, be the same style figure (in this case, one with a chiasmus-like structure). Depending on how emotionally involved a reader is and how strong the subconscious desire is to be moved by emotive literary style fragments, the top-down simulation can have a slight, medium, or robust effect.

In the first case it was predicted that the simulation will not stay in consciousness long, receding back almost immediately into long-term memory to await further activation in future reading scenarios. In case two, it was predicted that the simulation will dwell, and may even threaten to override incoming data, before receding into long-term memory, re-primed for possible future activation. In case three, it was predicted that the style figure in question would attain full consciousness with the real potential of overriding the actuality of the physical linguistic input on the page. It was pointed out that this process of overriding the semantic contact is something that has been attested in many experiments in discourse processing studies. Furthermore, such concepts as “expectations”, “set”, “anticipation”, “scenario mapping”, “inferencing” and “implicature” are all established models from either cognitive psychology or linguistics, which can all play a significant meaning-making role during acts of reading and listening. They also support the general precepts of simulation and the idea that concept-driven output can complement or even override stimulus-driven input depending on the situational factors.

Emotion will almost certainly play a significant role in all this literary discourse processing activity too. LeDoux (1998: 164), for example, has cogently argued that there are two routes to emotive processing: a longer route, which he calls the high road, and a short route, the low road. Unlike the “high road”, the short “quick and dirty” journey bypasses a lot of higher cortical processing. This means that emotive response can come into being without the higher cognitive appraisal of such an emotion initially having taken place. Within a framework of literary discourse processing, this has been referred to as “affective cognition”, as opposed to “cognitive emotion”, which takes the longer route (see Burke, 2011: 156–160).

Following Zeman et al. (2013) and Jacobs (2015a), brain activity generated by initial encounters with such style figures must be predominantly processed in the left hemisphere (Zeman) or the right hemisphere (Jacobs). In which half of the brain most activity takes place when a non-consciously remembered style figure, such as a chiasmus, is non-consciously activated and moved out of storage and into consciousness is unknown. Following the above mentioned empirical observations it is likely to primarily involve the left hemisphere.

In addition to rhetorical schemes it has been noted that poetic meter is ideally suited for neuro-literary investigation, not least because both schemes and meter share music-like minimalist structures (Burke, 2013: 213). The iambic, trochaic, anapestic or dactylic rhythms of neural processes that underlie the neuroaesthetics of prose and poetry are waiting to be charted. Rhetorical schemes and poetic meter harbor the potential to take literature out of its current neurobiological indiscernible and imperceptible world and into a perceptual, discernable and indeed measurable world, thus placing it alongside music, art and dance, the mainstay of current neuroaesthetic study. The neuroaesthetic potential of poetic meter was hypothesized when Turner and Pöeppel (1983: 303) noted that “poetry presents to the brain a system which is temporally and rhythmically hierarchical, as well as linguistically so, and therefore matched to the hierarchical organization of the brain itself”. It is these discrete musical, rhythmical literary structures like meter and style figure schemes (appropriately termed “devices” by Skov, 2009: 13) that will help neuroaesthetics enter and record the cortical world of literary discourse processing.

## Conclusion

In this article an attempt has been made to explore why it might be that neuroaesthetics tends not to engage with literature. Thereafter, and of far greater importance, the question was addressed as to how this might best be resolved. This discussion involved a general survey of the pitfalls, parameters and prospects of a neuroaesthetics of prose fiction. The core aim of this survey article has been to motivate, stimulate and invigorate thinking among neuroscientists as to how prose fiction might be better integrated into the canon of neuroaesthetics. From the perspective of research design and methodology, literary and rhetorical hypotheses should be tested in the laboratories of cognitive science. A mixed methods approach should be adopted at all times. This will entail on the one hand structural literary methods (like those found in narrative theory, classical rhetoric and literary stylistics) and neurobiological methods (like the measuring of pupil size, heart rate and galvanic skin responses, as well as fMRI and EEG scanning procedures). The neuroaesthetics of prose fiction needs humanists and scientists working together in a dedicated and concerted effort in order to make up the ground it has lost in the past decade to its sister arts of music, painting and dance. Everything is to be gained. Calliope is silently willing us not to disappoint her.

## Conflict of Interest Statement

The author declares that the research was conducted in the absence of any commercial or financial relationships that could be construed as a potential conflict of interest.
